# Parametric Analysis of Thick FGM Plates Based on 3D Thermo-Elasticity Theory: A Proper Generalized Decomposition Approach

**DOI:** 10.3390/ma16041753

**Published:** 2023-02-20

**Authors:** Mohammad-Javad Kazemzadeh-Parsi, Amine Ammar, Francisco Chinesta

**Affiliations:** 1 LAMPA & ESI Group Chair, Arts et Metiers Institute of Technology, 49035 Angers, France; 2 PIMM Lab & ESI Group Chair, Arts et Metiers Institute of Technology, 75013 Paris, France

**Keywords:** proper generalized decomposition, thermo-elasticity, a priori model order reduction, functionally graded material, thick plates

## Abstract

In the present work, the general and well-known model reduction technique, PGD (Proper Generalized Decomposition), is used for parametric analysis of thermo-elasticity of FGMs (Functionally Graded Materials). The FGMs have important applications in space technologies, especially when a part undergoes an extreme thermal environment. In the present work, material gradation is considered in one, two and three directions, and 3D heat transfer and theory of elasticity equations are solved to have an accurate temperature field and be able to consider all shear deformations. A parametric analysis of FGM materials is especially useful in material design and optimization. In the PGD technique, the field variables are separated to a set of univariate functions, and the high-dimensional governing equations reduce to a set of one-dimensional problems. Due to the curse of dimensionality, solving a high-dimensional parametric problem is considerably more computationally intensive than solving a set of one-dimensional problems. Therefore, the PGD makes it possible to handle high-dimensional problems efficiently. In the present work, some sample examples in 4D and 5D computational spaces are solved, and the results are presented.

## 1. Introduction

The new class of composite materials in which the mechanical properties evolve continuously in space is known as Functionally Graded Materials (FGM). In traditional composite materials, the mechanical properties evolve sharply at the interface of different phases, and this leads to high interfacial stresses and in many times the origin of failure. In contrast, the gradual variation of material properties in FGM materials prevents from such behaviors [1]. This feature makes the FGM the ideal choice in severe thermal environments and makes it possible to have a part with the pure metallic phase at one point to satisfy strength and the pure ceramic phase at another point to resist again high temperature [2]. The design of FGM materials consists of an accurate determination of spatial distribution of concentration (or volume fraction) of constituents. Here, different models are used to describe the materials distributions over the plate volume. These models are based on one or more controlling parameters that determine the materials distributions. After that, a parametric analysis is needed to assess the effect of these material distribution parameters on the mechanical performance of the whole component. Therefore, parametric study is one important step in the optimum design of FGM parts [3]. Recent technologies use additive manufacturing to produce FGM materials [4], and consequently, the FGM parts with multi-directional material variations are realistic today.

The bending behavior of thick or moderately thick plates is considerably affected by shear deformations, especially when complex materials such as laminated composites or FGM materials are considered. All plate bending theories such as Kirchhoff–Love plate theory or high-order plate theories assume a prespecified displacement distribution along the plate thickness. Such assumptions are good enough in thin plates or in the case of homogeneous or isotropic materials. However, when thick plates are considered or when complex materials with non-homogeneous or anisotropic characteristics are considered, the plate theories (classical and higher order) suffer from low accuracy. Therefore, the most general approach to consider all strains is using the continuum theory of elasticity [5,6]. The traditional computational techniques such as the finite element method are costly in 3D elasticity analysis of plate structures. It would be the worst in parametric analysis in which a full 3D analysis should be performed for any values or combinations of material parameters [3].

In the present work, to overcome the computational cost of a full parametric analysis, the PGD (Proper Generalized Decomposition) is used. The PGD makes it possible to overcome the curse of dimensionality and among other things allows solving a high-dimensional parametric problem and avoiding computational explosion. The basic idea behind of the PGD is to represent a multivariate function as a tensor product of a set of functions in lower dimensions. In addition, the PGD proposes a systematic and versatile procedure to obtain these low-dimensional functions.

The PGD was proposed for the first time to solve transient problems by space–time decomposition and avoiding the traditional step-by-step time marching. This space–time decomposition was originally suggested by Ladeveze and his coworkers within the framework of the LATIN strategy to develop a non-incremental transient solver [7,8]. Then, the first version of the PGD has been proposed in [9,10]. It was only this new version that makes it possible to deal with multidimensional problems in high-dimensional spaces. The method has been successfully applied in the first phase of its development for the kinetic theory modeling of complex fluids considering the high number of configuration space dimensions [9,10]. This situation particularly appears in the kinetic theory modeling of molten polymers or in bead–spring–chain modeling of polymer suspension. After that, the PGD technique was successfully applied in many different fields of science and engineering. Here, a few applications of PGD are mentioned just for a quick review. For instance, the PGD strategy was applied in the transient degradation of plastic materials [11]. It also was used to solve the so-called chemical master equation [12] and the problems involving Brownian configuration fields [13]. Heat transfer, thermal stress and residual stress problems in the forming of composite materials are other applications of this technique [14]. It was also used to study stochastic problems [15,16]. However, one of its most appealing uses concerns the fast and efficient solution of problems involving plate or shell structures. In such cases, the 3D field functions are decomposed into a tensor product of functions defined in the plane directions (say f(x,y)) and functions defined in the normal direction (say g(z)). Therefore, it make it possible to solve a full 3D problem with a computational complexity equivalent to a 2D problem [17]. The successful application of such a strategy was reported in [18] considering composite shell structures. Other applications considering a high order of interpolation in the thickness direction were reported in [19,20]. The in-plane and out-of-plane separation strategy were used recently in [21] considering a high-resolution discretization to capture the effects of ply thicknesses on the laminate composite stiffness. The application of PGD in thick plate elasticity analysis and the optimum material distribution design of FGM materials considering multi-directional material gradation was considered recently in [22]. The PGD was also used in non-regular or complex domains using NURBS geometry description and multipatch strategy [23,24].

In the current research, the parametric analysis of thermo-elastic thick plates consisting of FGMs is made based on the continuum theory of thermo-elasticity. Material gradation is considered in one, two and three directions of physical space. A model with some controlling parameters is considered to define the material distribution (volume fraction) of constituents over the physical space. A micromechanical model is used to obtain local mechanical properties based on the volume fractions. The PGD technique is used for solving the parametric problem in a high-dimensional computational space by representing the field variables as a tensor product of single variate functions in any direction of the computational space. In fact, this approach avoids repeated simulations to evaluate the effect of the material parameters. In other words, the material parameters are treated as extra dimensions of a high-dimensional computational domain.

The domain of application of the present technique is all the engineering and technology domains taking advantage of metamaterials, in particular in the domain of space structures, energy, insulation, barrier properties, etc. Our technology is not a new procedure for making that but rather a procedure that allows exploring the parametric domain in a very efficient way to find the optimal parameters determining the spatial distribution of the functionalities.

In the rest of this paper, the problem description as well as strong and weak forms of governing partial differential equations are presented in the next section. Then, the basic idea of separated representation is explained. It is followed with a concise description of PGD in thermo-elastic analysis. Four numerical examples are solved to show the potentials of PGD in the parametric analysis of FGM materials. An appendix is added to explain how a separated representation of a known field function in a high-dimensional space can be obtained. Finally, the paper is summarized in the conclusions.

## 2. Problem Description and Governing Equations

Consider the steady state and static thermo-elastic deformation occurring in a thick or moderately thick plate made of FGM material under thermal and mechanical loading. In such cases, the plate bending theories suffer from a lack of accuracy due to the importance of shear deformations and the complex behavior of FGM materials. The direct use of continuum theories is inevitable in such cases to obtain accurate solutions. The governing equations regarding heat transfer and thermo-elastic deformations and their weak forms are described in this section.

**Figure 1:**
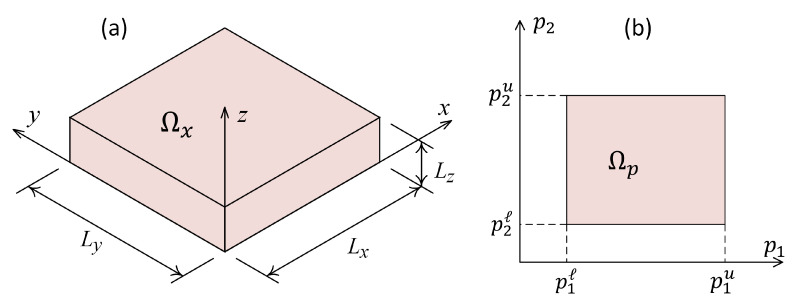
(**a**) Schematic representation of physical space $\Omega_{x}$, its coordinate system and dimensions; (**b**) Schematic representation of parametric space $\Omega_{p}$ considering two material parameters $p_{1}$ and $p_{2}$.

Figure 1a shows a schematic representation of the physical space, $\Omega_{x}$, coordinate system and its dimensions. The physical space is a three-dimensional space, $\Omega_{x}\in R^{3}$. In addition to physical space, there is another space that is called here a parametric space, $\Omega_{p}$. It defines the space of extra parameters that describe the way the constitutive materials are distributed over the physical space. The number of dimensions of the parametric space, $\Omega_{p}$, depends on the number of extra parameters that define the parametric study. For instance, Figure 1b shows a schematic representation of the parametric space considering two material distribution parameters $p_{1}$ and $p_{2}$.

The union of physical space, $\Omega_{x}$, and parametric space, $\Omega_{p}$, is called computational space, $\Omega =\Omega_{x}\cup \Omega_{p}$, where $\Omega \in R^{N_{D}}$. The number of dimensions of computational space, $N_{D}$, is 3 plus the number of material distribution parameters. Any point of the computational space, x∈Ω, is defined as follows:

(1) $$x=\left(x_{1},x_{2},x_{3},x_{4},\cdots ,x_{N_{D}}\right)\equiv \left(x,y,z,p_{1},p_{2},\cdots \right)\;,$$

where the first three coordinates $x_{1}$ to $x_{3}$ correspond to physical coordinates *x* to *z*, and extra coordinates $x_{4}$ and beyond correspond to material parameters $p_{1}$ and beyond.

The governing equation regarding conductive heat transfer in a non-homogeneous and isotropic media considering distributed body heat generation s(x) is as follows:

(2) $$\frac{\partial }{\partial x_{1}}\left(k\frac{\partial T}{\partial x_{1}}\right)+\frac{\partial }{\partial x_{2}}\left(k\frac{\partial T}{\partial x_{2}}\right)+\frac{\partial }{\partial x_{3}}\left(k\frac{\partial T}{\partial x_{3}}\right)+s=0\;,$$

where T(x) is the temperature field and k(x) is the thermal conductivity field. In fact, these functions are defined in the computational space, Ω, and x∈Ω.

A micromechanical model is needed to make an approximation for the thermal conductivity, k(x), at each point based on the concentration of constitutive materials (volume fractions) and also the thermal behavior of constituents. More explanations regarding the micromechanical model are given later.

The static deformations of a 3D continuum media under the body forces $b_{1}$, $b_{2}$ and $b_{3}$ are governed by the static equilibrium equations as follows:

(3) $$\begin{array}{c} \frac{\partial \sigma_{11}}{\partial x_{1}}+\frac{\partial \sigma_{12}}{\partial x_{2}}+\frac{\partial \sigma_{13}}{\partial x_{3}}+b_{1}=0\;,\\ \frac{\partial \sigma_{21}}{\partial x_{1}}+\frac{\partial \sigma_{22}}{\partial x_{2}}+\frac{\partial \sigma_{23}}{\partial x_{3}}+b_{2}=0\;,\\ \frac{\partial \sigma_{31}}{\partial x_{1}}+\frac{\partial \sigma_{32}}{\partial x_{2}}+\frac{\partial \sigma_{33}}{\partial x_{3}}+b_{3}=0\;, \end{array}$$

where symbols σ with different indices refer to stress components. Considering non-homogeneous and isotropic deformations, the linear elastic stress–strain relation with the thermal effects is given by:

(4) $${\left[\sigma_{11},\sigma_{22},\sigma_{33},\sigma_{12},\sigma_{23},\sigma_{31}\right]}^{T}=C{\left[\epsilon_{11},\epsilon_{22},\epsilon_{33},\epsilon_{12},\epsilon_{23},\epsilon_{31}\right]}^{T}-B\tau \;,$$

where symbols ϵ show strain components and $\tau =T-T_{0}$ is the temperature rise with respect to stress-free temperature $T_{0}$. The coefficient matrix, C, and vector, B, are the elasticity matrix and thermo-elasticity vector, respectively.

Generally, the elasticity matrix, C, and thermo-elasticity vector, B, are functions of space coordinates and also material distribution parameters $x=x_{1},x_{2},\cdots ,x_{N_{D}}$. The details regarding basic concepts and derivations of C and B are available in many references such as [25]. Here, just for completeness, the relations for nonzero components of C and B in terms of engineering constants are given as follows for isotropic materials:

(5) $$\begin{array}{cc} C_{11}=C_{22}=C_{33} & =\frac{(1-\nu )E}{\left(1+\nu )(1-2\nu \right)}\;,\\ C_{44}=C_{55}=C_{66} & =\frac{E}{\left(1+\nu \right)}\;,\\ C_{12}=C_{13}=C_{23} & =\frac{\nu E}{\left(1+\nu )(1-2\nu \right)}\;,\\ B_{1}\;=B_{2}=\;B_{3}\; & =\frac{\alpha E}{\left(1-2\nu \right)}\;, \end{array}$$

where the engineering constants of Young modulus, E(x), Poisson ratio, ν(x), and coefficient of thermal expansion, α(x), are functions of x. Proper micromechanical models are also needed to approximate these values as functions of the volume fraction of the constituents and also their mechanical properties.

The strain–displacement relations for small deformations are as follows:

(6) $$\begin{array}{cc} \; & \epsilon_{11}=\frac{\partial u_{1}}{\partial x_{1}}\;,\;\epsilon_{22}=\frac{\partial u_{2}}{\partial x_{2}}\;,\;\epsilon_{33}=\frac{\partial u_{3}}{\partial x_{3}}\;,\\ \; & \epsilon_{12}=\frac{1}{2}\left(\frac{\partial u_{2}}{\partial x_{1}}+\frac{\partial u_{1}}{\partial x_{2}}\right)\;,\;\epsilon_{23}=\frac{1}{2}\left(\frac{\partial u_{3}}{\partial x_{2}}+\frac{\partial u_{2}}{\partial x_{3}}\right)\;,\;\epsilon_{31}=\frac{1}{2}\left(\frac{\partial u_{1}}{\partial x_{3}}+\frac{\partial u_{3}}{\partial x_{1}}\right)\;, \end{array}$$

where $u_{1}$, $u_{2}$ and $u_{3}$ are displacement components and symbols ϵ show different strain components.

Now, the weak forms of governing partial differential equations are needed to develop the numerical solution. Consider the heat equation given at Equation (2) and apply the weighted residual method to obtain the integral form and then apply the integration by parts formula to derive the weak form and the natural (or Neumann) boundary conditions as follows [25]:

(7) $$\begin{array}{c} \int_{\Omega_{x}}\left(k\frac{\partial T^{*}}{\partial x_{1}}\frac{\partial T}{\partial x_{1}}+k\frac{\partial T^{*}}{\partial x_{2}}\frac{\partial T}{\partial x_{2}}+k\frac{\partial T^{*}}{\partial x_{3}}\frac{\partial T}{\partial x_{3}}\right)d\Omega_{x}=\\ \int_{\Omega_{x}}T^{*}sd\Omega_{x}+\int_{\Gamma_{N}^{t}}T^{*}qd\Gamma \;, \end{array}$$

where $\Omega_{x}$ is the physical space and *q* denotes the prespecified external thermal flux acting on the natural boundary (or Neumann boundary), $\Gamma_{N}^{t}$. In this equation, $T^{*}$ is the first variation of the temperature field.

Use the weighted residual method and then perform the integration by parts to derive the weak forms of elasticity equations, Equation (3). After that, substitute Equations (4) and (6) to obtain the weak form and natural (or traction) boundary conditions as follows [25]:

(8) $$\begin{array}{c} \int_{\Omega_{x}}\left(\frac{\partial u_{1}^{*}}{\partial x_{1}}\left(C_{11}\frac{\partial u_{1}}{\partial x_{1}}+C_{12}\frac{\partial u_{2}}{\partial x_{2}}+C_{13}\frac{\partial u_{3}}{\partial x_{3}}-B_{1}\tau \right)+C_{44}\frac{\partial u_{1}^{*}}{\partial x_{2}}\left(\frac{\partial u_{2}}{\partial x_{1}}+\frac{\partial u_{1}}{\partial x_{2}}\right)+\right.\\ \left.C_{66}\frac{\partial u_{1}^{*}}{\partial x_{3}}\left(\frac{\partial u_{1}}{\partial x_{3}}+\frac{\partial u_{3}}{\partial x_{1}}\right)\right)d\Omega_{x}=\int_{\Gamma_{N}^{e}}u_{1}^{*}t_{1}d\Gamma +\int_{\Omega_{x}}u_{1}^{*}b_{1}d\Omega_{x}\;, \end{array}$$

(9) $$\begin{array}{c} \int_{\Omega_{x}}\left(C_{44}\frac{\partial u_{2}^{*}}{\partial x_{1}}\left(\frac{\partial u_{2}}{\partial x_{1}}+\frac{\partial u_{1}}{\partial x_{2}}\right)+\frac{\partial u_{2}^{*}}{\partial x_{2}}\left(C_{12}\frac{\partial u_{1}}{\partial x_{1}}+C_{22}\frac{\partial u_{2}}{\partial x_{2}}+C_{23}\frac{\partial u_{3}}{\partial x_{3}}-B_{2}\tau \right)+\right.\\ \left.C_{55}\frac{\partial u_{2}^{*}}{\partial x_{3}}\left(\frac{\partial u_{3}}{\partial x_{2}}+\frac{\partial u_{2}}{\partial x_{3}}\right)\right)d\Omega_{x}=\int_{\Gamma_{N}^{e}}u_{2}^{*}t_{2}d\Gamma +\int_{\Omega_{x}}u_{2}^{*}b_{2}d\Omega_{x}\;, \end{array}$$

(10) $$\begin{array}{c} \int_{\Omega_{x}}\left(C_{66}\frac{\partial u_{3}^{*}}{\partial x_{1}}\left(\frac{\partial u_{1}}{\partial x_{3}}+\frac{\partial u_{3}}{\partial x_{1}}\right)+C_{55}\frac{\partial u_{3}^{*}}{\partial x_{2}}\left(\frac{\partial u_{3}}{\partial x_{2}}+\frac{\partial u_{2}}{\partial x_{3}}\right)\right.\\ \left.\frac{\partial u_{3}^{*}}{\partial x_{3}}\left(C_{13}\frac{\partial u_{1}}{\partial x_{1}}+C_{23}\frac{\partial u_{2}}{\partial x_{2}}+C_{33}\frac{\partial u_{3}}{\partial x_{3}}-B_{3}\tau \right)\right)d\Omega_{x}=\int_{\Gamma_{N}^{e}}u_{3}^{*}t_{3}d\Gamma +\int_{\Omega_{x}}u_{3}^{*}b_{3}d\Omega_{x}\;, \end{array}$$

where $u^{*}$ in Equations (8)–(10) is the variation of the displacement fields in different directions. The surface tractions are shown by $t_{1}$, $t_{2}$ and $t_{3}$ on the natural (or traction) boundary $\Gamma_{N}^{e}$. The symbols *C* and *B* with different indices show nonzero components of matrix C and vector B. Three equations in Equations (8)–(10) have the same structure, and it is possible to rewrite them in a generic form as follows:

(11) $$\sum_{\mathrm{term}\;1}^{\mathrm{term}\;7}\int_{\Omega_{x}}\frac{\partial u_{a}^{*}}{\partial x_{b}}\frac{\partial u_{c}}{\partial x_{d}}C_{ef}d\Omega =\int_{\Gamma_{N}^{e}}u_{a}^{*}t_{a}d\Gamma +\int_{\Omega_{x}}u_{a}^{*}b_{a}d\Omega_{x}+\int_{\Omega_{x}}u_{a}^{*}B_{a}\tau d\Omega_{x},$$

where indices *a* to *f* are given in Table 1 for each term of the weak form in Equations (8)–(10). The main advantage of such a compressed form is that it facilitates the computer implementation and programming of the PGD technique.

In summary, the basic steps of thermo-elasticity analysis are: first, solving the weak form of heat equation given in Equation (7) and obtaining the temperature field *T*, and second, solving the weak form of elasticity equation given in Equation (11) and obtaining the displacement fields $u_{1}$, $u_{2}$ and $u_{3}$.

## 3. Field Function Separation

Consider the scalar field function g(x), x∈Ω, is defined in the computational space, $R^{N_{D}}\to R$. As explained before, the number of dimensions of the computational space, Ω, is shown by $N_{D}$. The function g(x) may be an unknown function such as temperature or displacements fields. On the other hand, the field g(x) may be a given (or known) function such as the thermal conductivity k(x) or the elasticity coefficients $C_{ef}\left(x\right)$. Nevertheless, in general, it is possible to separate (or decompose) the g(x) into a superposition of tensor product of low-dimensional functions. The result is called Separated Approximate Representation (SAR) of g(x) as follows:

(12) $$g\left(x\right)\approx g^{h}\left(x\right)=\sum_{i=1}^{N_{g}}g_{1i}\left(x_{1}\right)g_{2i}\left(x_{2}\right)g_{3i}\left(x_{3}\right)g_{4i}\left(x_{4}\right)\cdots \;,$$

where the superscript *h* in $g^{h}\left(x\right)$ indicates that this is an approximated representation because the number of terms in the summation, $N_{g}$, are finite. In Equation (12), the functions $g_{1i}\left(x_{1}\right)$, $g_{2i}\left(x_{2}\right)$, ⋯ are univariate functions in directions $x_{1}$, $x_{2}$, ⋯, respectively. Remember that the first three dimensions $x_{1}$ to $x_{3}$ refer to physical space *x* to *z* and the dimensions $x_{4}$ and beyond refer to parametric space $p_{1}$ and beyond.

Equation (12) states that the approximated field $g^{h}\left(x\right)$ is built up by the superposition of a set of functions; each of them is constructed by the product of 1D functions in each direction of computational space. In the PGD literature, these univariate functions are called modes.

We are seeking to solve the problem numerically. Therefore, the discrete form of the univariate functions $g_{1i}\left(x_{1}\right)$, $g_{2i}\left(x_{2}\right),\cdots$ is searched. One way is to express them by some predefined approximation functions (shape functions) and corresponding unknown coefficients (nodal values) as used in standard 1D finite element approximation as follows:

(13) $$g\left(x\right)=\sum_{i=1}^{N_{g}}M_{1}^{T}\left(x_{1}\right)g_{1i}M_{2}^{T}\left(x_{2}\right)g_{2i}\cdots =\sum_{i=1}^{N_{g}}\prod_{j=1}^{N_{D}}M_{j}^{T}\left(x_{j}\right)g_{ji},$$

where superscript *h* is dropped for simplicity. The vector $M_{j}\left(x_{j}\right)$ in Equation (13) contains the shape functions (or approximation functions) in terms of the *j*-th coordinate direction of computational space. The vector $g_{ji}$ contains the nodal values (or coefficients) associated with the *j*-th direction. In this equation, the index *i* shows the summation index and $N_{g}$ is the number of terms in the SAR of g(x).

The discretization of the univariate functions as given in Equation (13) is similar to finite element discretization of one-dimensional functions. The vector $M_{j}\left(x_{j}\right)$ is called the shape functions vector and the vector $g_{ji}$ is the nodal values vector. The simplest way to construct the shape functions $M_{j}\left(x_{j}\right)$ is to use the Lagrange interpolation functions of order one, which are piece-wise linear one-dimensional shape functions. However, in general, there are no restrictions, and any order of approximation can be used. Another possibility is to use a different order of approximations (or even special functions) in each direction to capture specific characteristics based on the physical behavior of the system.

In general, the generic function g(x) may be an available (or known) function or it may be an unknown function. If g(x) is a given (or known) function, the vectors $g_{ji}$ can be calculated via defining a minimization problem and trying to minimize an error function iteratively. The details of this process are explained in detail in Appendix A.

If the field function g(x) is unknown, e.g., displacement or temperature distributions, the governing equations must be used to obtain the vectors $g_{ji}$. The PGD technique proposes an approach for solving governing equations and obtaining unknown coefficients. The technique is described in detail in Section 4.

## 4. Proper Generalized Decomposition

The main steps of thermo-elastic analysis consist of solving heat equation at first to obtain the temperature field and then solving elasticity equations to obtain displacement fields. The PGD technique is utilized here to resolve these equations. A more detailed procedure for the whole process is presented below:

1. Input the material properties of the base materials and the volume fraction distribution as a function defined on the computational space Ω.
2. Apply a suitable micromechanical model to evaluate thermal conductivity distribution, k(x), over Ω (will be described later in Equation (30)).
3. Create an SAR for the thermal conductivity (using Appendix A).
4. Create an SAR for body heat generation and surface heat fluxes (using Appendix A).
5. Use the PGD technique to solve the heat equation, Equation (7), and obtain an SAR for the temperature field (using Section 4.1).
6. Use suitable micromecanical models to calculate the distribution of *E*, ν and α over Ω (will be described later in Equations (26), (27) and (31)).
7. Calculate the distribution of all nonzero elements of matrix C and vector B over Ω (using Equation (5)).
8. Create an SAR for each nonzero element of matrix C and vector B (using Appendix A).
9. Create an SAR for all body forces and surface tractions (using Appendix A).
10. Use the PGD technique to solve the elasticity equations, Equation (11), and obtain an SAR for each displacement component (using Section 4.2).

Regarding steps 3, 4, 8 and 9, it must be mentioned that the thermal conductivity, *k*, surface heat flux *q*, elasticity matrix C and thermo-elasticity vector B are obtainable functions on the computational spaces. Therefore, the construction of SAR for all of these field functions can be made by using the procedure given in Section 3 and Appendix A. The following relations express these separated representations:

(14) $$k\left(x\right)=\sum_{i=1}^{N_{k}}\prod_{j=1}^{N_{D}}M_{j}^{T}\left(x_{j}\right)k_{ji},$$

(15) $$C_{ef}\left(x\right)=\sum_{i=1}^{N_{C_{ef}}}\prod_{j=1}^{N_{D}}M_{j}^{T}\left(x_{j}\right)C_{efji},$$

(16) $$B_{e}\left(x\right)=\sum_{i=1}^{N_{B_{e}}}\prod_{j=1}^{N_{D}}M_{j}^{T}\left(x_{j}\right)B_{eji},$$

(17) $$q\left(x\right)=\sum_{i=1}^{N_{q}}\prod_{j=1}^{N_{D}-1}M_{j}^{T}\left(x_{j}\right)q_{ji},$$

(18) $$t_{a}\left(x\right)=\sum_{i=1}^{N_{t}}\prod_{j=1}^{N_{D}-1}M_{j}^{T}\left(x_{j}\right)t_{aji},$$

where *N* with different indices shows the number of terms in SAR of different fields and the vectors $k_{ji}$, $C_{efji}$, ⋯ are coefficient vectors corresponding to the *i*-th term in the *j*-th coordinate (see Section 3 and Appendix A).

Note that the surface heat flux q(x) and surface traction $t_{a}\left(x\right)$ are defined on the boundary surfaces of the physical space ($R^{N_{D}-1}$). These functions also appeared in surface integrals on natural boundaries.

Steps 5 and 10 of the foregoing procedure are talking about the PGD solution of governing equations. The following two subsections explain the PGD technique for solving both heat and elasticity equations separately.

### 4.1. PGD Solution of Heat Equation

Here, the PGD technique is used for constructing an SAR for temperature distribution by solving the weak form given in Equation (7). Consider the temperature field T(x) is depicted as the successive separated form:

(19) $$T\left(x\right)=\sum_{i=1}^{n}\prod_{j=1}^{N_{D}}M_{j}^{T}\left(x_{j}\right)T_{ji}\;.$$

Suppose that the first (n−1) terms in Equation (19) are known, and finding the next term, *n*, is seeking. The above equation is rearranged as follows:

(20) $$T\left(x\right)=\sum_{i=1}^{n-1}\prod_{j=1}^{N_{D}}M_{j}^{T}\left(x_{j}\right)T_{ji}+\prod_{j=1}^{N_{D}}M_{j}^{T}\left(x_{j}\right)T_{jn}\;.$$

To explain it more, assume that the vectors $T_{ji}$ regarding terms i∈{1,2,⋯,n−1} are already known and the coefficient vector $T_{jn}$ regarding the last term, *n*, should be obtained in such a way that the temperature field in Equation (20) satisfies the weak form. The first variation of the temperature, $T^{*}$, taking into account that the first n−1 terms are known and consequently their variations vanish, reads:

(21) $$T^{*}=\sum_{d=1}^{N_{D}}M_{d}^{T}\left(x_{d}\right)T_{dn}^{*}\prod_{\begin{array}{c} j=1\\ j\neq d \end{array}}^{N_{D}}M_{j}^{T}\left(x_{j}\right)T_{jn}\;.$$

Using the chain rule to differentiate from *T* and $T^{*}$ (Equations (20) and (21)) with respect to $x_{a}$, we have:

(22) $$\frac{\partial T}{\partial x_{a}}=\sum_{i=1}^{n-1}\frac{\partial M_{a}^{T}}{\partial x_{a}}T_{ai}\prod_{\begin{array}{c} j=1\\ j\neq a \end{array}}^{N_{D}}M_{j}^{T}T_{ji}+\frac{\partial M_{a}^{T}}{\partial x_{a}}T_{an}\prod_{\begin{array}{c} j=1\\ j\neq a \end{array}}^{N_{D}}M_{j}^{T}T_{jn}\;,$$

(23) $$\frac{\partial T^{*}}{\partial x_{a}}=\frac{\partial M_{a}^{T}}{\partial x_{a}}T_{an}^{*}\prod_{\begin{array}{c} j=1\\ j\neq a \end{array}}^{N_{D}}M_{j}^{T}T_{jn}+\sum_{d=1}^{N_{D}}M_{d}^{T}T_{dn}^{*}\frac{\partial M_{a}^{T}}{\partial x_{a}}T_{an}\prod_{\begin{array}{c} j=1\\ j\neq d\\ j\neq a \end{array}}^{N_{D}}M_{j}^{T}T_{jn}\;.$$

By substituting Equations (22) and (23) in Equation (7), a system of equations in terms of unknown coefficients $T_{jn}$ is obtained. When this system is solved and the modes regarding each univariate function are obtained, the *n*-th term of Equation (20) will be available. The same process could be repeated in the same way to obtain subsequent terms.

The foregoing system of algebraic equations is nonlinear, and an iterative technique can be utilized to solve it. The simplest and the most widely used approach is the method of fixed point iterations [26]. In each iteration of this iterative approach, it is assumed that only one univariate function is unknown, and the rest of them are known. This assumption linearizes the algebraic system of equations and makes it possible to solve them directly. After that, in the next iteration, another univariate function is considered unknown and the process repeats. This process operates iteratively and makes it possible to resolve the nonlinear system of algebraic equations easily. A stopping criterion is needed in general to stop the fixed point iterations.

It should be mentioned that after obtaining the *n*-th term, as explained above, the process repeats to obtain subsequent terms. In the PGD literature, this process is called the enriching process. A stopping criterion is also needed in this level to terminate the enriching process when an accuracy level is achieved.

To develop a computer code for implementing the PGD technique, two nested loops are needed. The first loop (outer one) is called the enrichment loop, and its goal is to add more terms to Equation (19) to enrich the solution. In the second loop (inner loop), the fixed point technique is applied to solve the nonlinear algebraic equations [26].

### 4.2. PGD Solution of Elasticity Equations

The PGD technique for solving a heat equation was described in Section 4.1. The same procedure should be followed to obtain the displacement components $u_{a}\left(x\right)$ using the weak form of elasticity equations given in Equation (11). It must be noted that the temperature rise, τ, in Equation (11) is available in this step because the heat equation is already solved.

Consider a separated form of the displacement $u_{a}$ (see Equation (12)). Without loss of generality, suppose the terms from i=1 to i=(n−1) of the separated form are known and finding the next term, i=n, is desirable. In fact, this is a step-by-step process, and at each step, a new term is added to the previously computed ones. The separated form of $u_{a}$ is as follows:

(24) $$u_{a}\left(x\right)=\sum_{i=1}^{n-1}\prod_{j=1}^{N_{D}}M_{j}^{T}\left(x_{j}\right)u_{aji}+\prod_{j=1}^{N_{D}}M_{j}^{T}\left(x_{j}\right)u_{ajn}\;.$$

To explain this more, suppose that the vectors $u_{aji}$, i∈{1,2,⋯n−1} are known (they were computed before) and the vectors $u_{ajn}$ are unknown and must be found using governing equations. Perform the first variation on Equation (24) while keeping in mind that the first summation is known; the result is as follows:

(25) $$u_{a}^{*}\left(x\right)=\sum_{d=1}^{N_{D}}M_{d}^{T}\left(x_{d}\right)u_{adn}^{*}\prod_{\begin{array}{c} j=1\\ j\neq d \end{array}}^{N_{D}}M_{j}^{T}\left(x_{j}\right)u_{ajn}\;.$$

Differentiate from Equations (24) and (25) and substitute the results in Equation (11) to have a system of equations in terms of unknown vectors $u_{ajn}$. The fixed point iterative method is again used here to solve these equations. As explained before in Section 4.1, in each iteration of the fixed-point algorithm, one mode is considered as unknown, and the other ones are considered known. This assumption linearizes the equations, and a direct system solver can be used. In the next fixed-point iteration, another mode is considered unknown, and the process repeats. This algorithm continues until satisfying a stopping criterion.

As explained before, after the *n*-th term is obtained, the same procedure repeats to obtain the next term n+1. Therefore, this step-by-step enrichment process improves the solution by adding more terms to the summation in Equation (24). A termination criterion is needed to terminate the enrichment process as soon as a level of accuracy is achieved.

The procedure which is explained above is the standard PGD algorithm. More details regarding its theoretical background and practical implementation (or developing a computer code) are available in the literature. For instance, interested readers are referred to [26,27,28].

## 5. Numerical Examples

Four sample examples are presented here to show the potentials and applicability of the PGD method for the parametric analysis of FGM materials. To represent the material distribution over the physical domain $\Omega_{x}$, a model is needed. For instance, power law, sigmoid, exponential and polynomial distributions are some well-known examples that describe how the constitutive materials are distributed over the physical space. Generally, the material distribution models contain some controlling parameters that describe the materials distribution. These parameters affect directly the mechanical performance of the FGM part. In the present section, it is shown by solving some numerical examples that the PGD technique can overcome the curse of dimensionality and efficiently handle parametric studies in high-dimensional spaces.

In all of the following examples, an FGM plate consists of a metallic matrix (Monel, 70Ni-30Cu) reinforced by spherical ceramic particles (Zirconia, ZrO${}_{2}$) distributed randomly over the volume. The mechanical/thermal characteristics of each base material are presented in Table 2 [29].

A micro-mechanical model is needed to evaluate the effective local material properties. Here, the bulk modulus of elasticity, κ, and shear modulus, μ, are estimated by utilizing the Mori–Tanaka material model [30,31] as given in the Equations (26) and (27). In all four numerical examples in the present work, the subscripts *m* and *c* are referring to the metallic phase and ceramic phase, respectively.

(26) $$\frac{\kappa -\kappa_{m}}{\kappa_{c}-\kappa_{m}}=\frac{V_{c}}{1+\left(1+V_{c}\right)\frac{\kappa_{c}-\kappa_{m}}{\kappa_{m}+f_{\kappa }}},$$

(27) $$\frac{\mu -\mu_{m}}{\mu_{c}-\mu_{m}}=\frac{V_{c}}{1+\left(1+V_{c}\right)\frac{\mu_{c}-\mu_{m}}{\mu_{m}+f_{\mu }}},$$

where $V_{c}$ is the volume fraction of ceramic phases. The volume fraction of the metallic phase is $V_{m}=1-V_{c}$. The functions $f_{\kappa }$ and $f_{\mu }$ are defined as follows:

(28) $$f_{\kappa }=\frac{4}{3}\mu_{m},$$

(29) $$f_{\mu }=\frac{\mu_{m}\left(9\kappa_{m}+8\mu_{m}\right)}{6(\kappa_{m}+2\mu_{m})}\;.$$

The local effective thermal conductivity, *k*, is estimated using the Hatta–Taya model [32] as follows:

(30) $$\frac{k-k_{m}}{k_{c}-k_{m}}=\frac{V_{c}}{1+\left(1+V_{c}\right)\frac{k_{c}-k_{m}}{3k_{m}}}\;.$$

The effective thermal expansion coefficient α is approximated as follows [33,34,35,36]:

(31) $$\frac{\alpha -\alpha_{m}}{\alpha_{c}-\alpha_{m}}=\frac{\frac{1}{\kappa }-\frac{1}{\kappa_{m}}}{\frac{1}{\kappa_{c}}-\frac{1}{\kappa_{m}}}\;.$$

### 5.1. Example 1

As the first numerical example, a problem with an available analytic solution is selected to be able to compare the PGD results with the exact solution. The power law is used here to describe the material distribution over the plate thickness in the $x_{3}$ direction. The power law consists of one material distribution parameter and the parametric space, $\Omega_{p}\in R$, is one-dimensional space. Therefore, in this example, the computational space, $\Omega \in R^{4}$, is 4D space, and the PGD technique converts the original 4D problem to a sequence of low-dimensional (1D) problems.

In the present example, the bottom surface of the plate ($x_{3}=0$) is considered as a pure metallic phase ($V_{c}=0$) and the upper surface ($x_{3}=L_{z}$) is considered as a pure ceramic phase ($V_{c}=1$). The material gradation is considered uni-directional through plate thickness based on the power law as follows:

(32) $$V_{c}\left(x\right)={\bar{x_{3}}}^{p}={\left(\bar{x_{3}}\right)}^{x_{4}},$$

where the parameter *p* is the material distribution parameter that describes the distribution of the ceramic phase through the thickness in the $x_{3}$ direction. In Equation (32), ${\bar{x}}_{3}$ is the non-dimensional coordinate in the $x_{3}$ direction as defined below:

(33) $${\bar{x}}_{1}=\frac{x_{1}}{L_{x}},\;\;\;\;{\bar{x}}_{2}=\frac{x_{2}}{L_{y}},\;\;\;\;{\bar{x}}_{3}=\frac{x_{3}}{L_{z}}.$$

Thermal boundary conditions consist of the following prespecified temperature distribution on the upper face of the plate at ${\bar{x}}_{3}=1$ and zero value at all other faces:

(34) $$T\left(x\right)=\hat{T}\sin\left(\pi {\bar{x}}_{1}\right)\sin\left(\pi {\bar{x}}_{2}\right),\;\;\mathrm{on}\;\mathrm{face}\;\;{\bar{x}}_{3}=1,$$

where $\hat{T}$ is the temperature amplitude. Regarding the boundary conditions of the elasticity problem, consider that the four sides of the plate are restrained by simple supports. In detail, we have:

(35) $$\begin{array}{cc} \; & u_{2}=u_{3}=0,\;\;\mathrm{on}\;\mathrm{faces}\;\;{\bar{x}}_{1}=0\;\mathrm{and}\;{\bar{x}}_{1}=1,\\ \; & u_{1}=u_{3}=0,\;\;\mathrm{on}\;\mathrm{faces}\;\;{\bar{x}}_{2}=0\;\mathrm{and}\;{\bar{x}}_{2}=1. \end{array}$$

To solve the reduced 1D problems, the standard finite element technique using linear two-node elements is utilized. The number of nodes in each direction are selected here as 61, 61, 41, and 16, respectively, in directions $x_{1}$, $x_{2}$, $x_{3}$, and $x_{4}$. Both thermal and elasticity problems are solved considering 20 enrichment steps.

**Figure 2:**
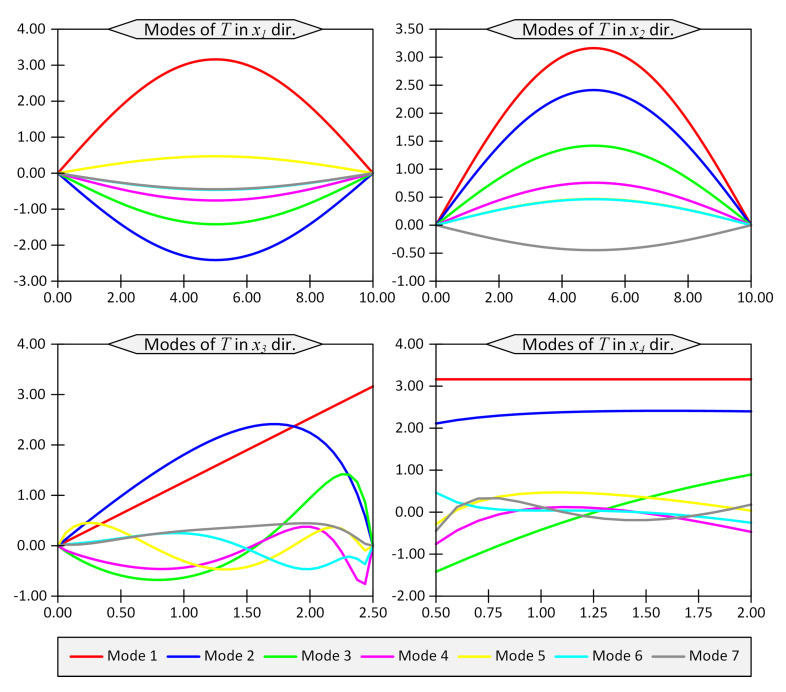
First 7 modes of temperature field *T* in directions $x_{1}$ to $x_{4}$ for Example 1 for thickness ratio $L_{x}/L_{z}=4$.

**Figure 3:**
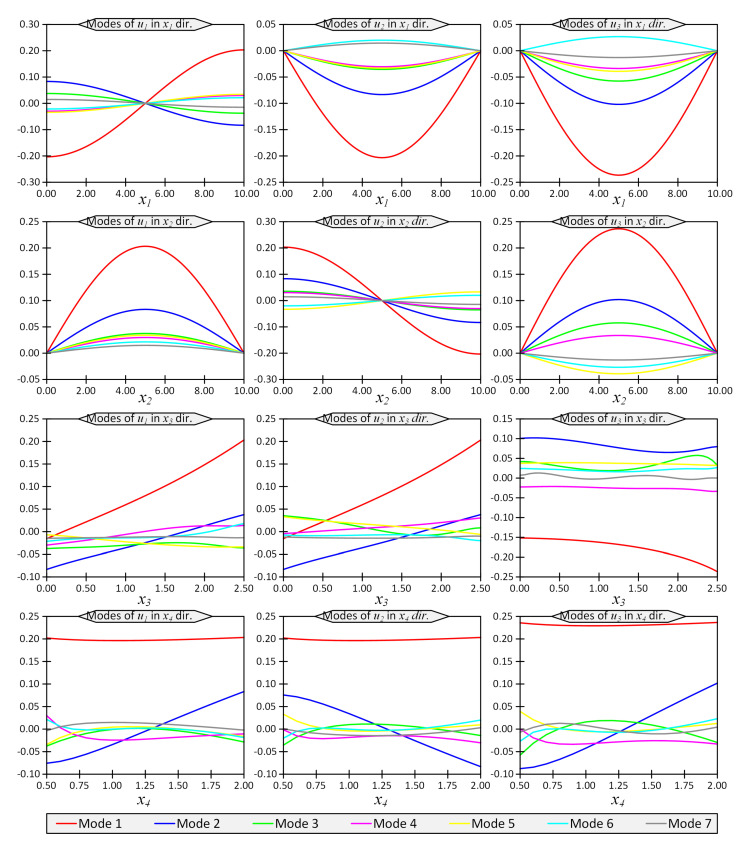
First 7 modes of $u_{1}$, $u_{2}$ and $u_{3}$ in directions $x_{1}$ to $x_{4}$ for Example 1 for thickness ratio $L_{x}/L_{z}=4$.

The first seven modes regarding the temperature field, *T*, and the vertical displacement, $u_{3}$, are shown in Figure 2 and Figure 3, respectively. The horizontal axes in these figures represent the coordinates in the computational space, while the vertical axes show the modes. In other words, vertical axes do not have a clear physical meaning, while their product would produce the physical quantities such as temperature or displacements.

To present the results and to compare them with the reference solution given in [29], the non-dimensional temperature, $\bar{T}$, displacement, ${\bar{u}}_{3}$, and stress, ${\bar{\sigma }}_{11}$ are defined as follows [29]:

(36) $$\bar{T}=\frac{T}{\hat{T}},\;\;\;\;{\bar{u}}_{3}=\frac{u_{3}}{10^{-6}\hat{T}L_{x}},\;\;\;\;{\bar{\sigma }}_{11}=\frac{\sigma_{11}}{10^{3}\hat{T}}.$$

Considering square plates, $L_{x}=L_{y}$, with two width-to-thickness ratios of $L_{x}/L_{z}=4$ (thick plate) and $L_{x}/L_{z}=10$ (moderately thick plate), the non-dimensional quantities $\bar{T}$, ${\bar{u}}_{3}$ and ${\bar{\sigma }}_{11}$ are given in Table 3 for the present PGD solution and also for the reference analytic solution given in [29]. In this table, the results are given at certain points at the center of the plate at ${\bar{x}}_{1}={\bar{x}}_{2}=0.5$ and different ${\bar{x}}_{3}$, as indicated in Table 3. The values in the parentheses show the relative percentage error of the present PGD solution with respect to the reference closed-form solution. Table 3 shows that the PGD solutions are in high agreement with the analytical solutions.

**Figure 4:**
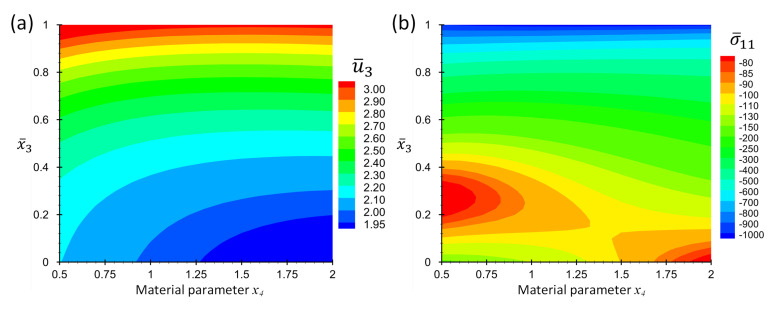
Contour plots of (**a**) non-dimensional displacement, ${\bar{u}}_{3}$, and (**b**) non-dimensional stress, ${\bar{\sigma }}_{11}$, at location ${\bar{x}}_{1}={\bar{x}}_{2}=0.5$ and in terms of ${\bar{x}}_{3}$ and $x_{4}$ for Example 1 for thickness ratio $L_{x}/L_{z}=4$.

**Figure 5:**
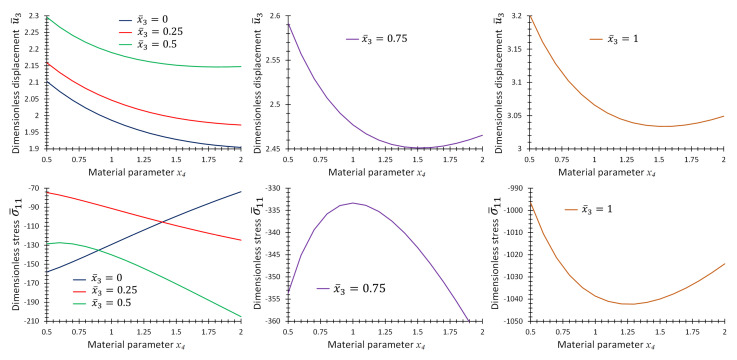
Graphs of non-dimensional quantities ${\bar{u}}_{3}$ and ${\bar{\sigma }}_{11}$, at location, ${\bar{x}}_{1}={\bar{x}}_{2}=0.5$, for different ${\bar{x}}_{3}$ in terms of material parameter $x_{4}$ for Example 1 for thickness ratio $L_{x}/L_{z}=4$.

Consider the case $L_{x}/L_{z}=4$. Figure 4 shows the contour plots of non-dimensional displacement, ${\bar{u}}_{3}$, and stress, ${\bar{\sigma }}_{11}$, for a point at location ${\bar{x}}_{1}={\bar{x}}_{2}=0.5$ in terms of the coordinate ${\bar{x}}_{3}$ and material parameter *p*. Figure 5 shows the graph format of the same data in Figure 4 to make it possible for the readers to extract the results accurately.

### 5.2. Example 2

In the this sample problem, an extension of the first example in Section 5.1 is considered. Here, the thermal boundary condition of the upper face is changed to the heat flux boundary condition as described in the following equation:

(37) $$q=1000,\;\;\;\mathrm{on}\;\mathrm{face}\;\;\;{\bar{x}}_{3}=1\;.$$

All other boundary conditions regarding the structural and thermal problems are identical with the previous example in Section 5.1. The same material distribution model is also used. The problem is solved using PGD with all conditions similar to Example 1. For instance, the number of nodes for each univariate function and the number of enrichment steps are exactly the same as explained in Section 5.1.

**Figure 6:**
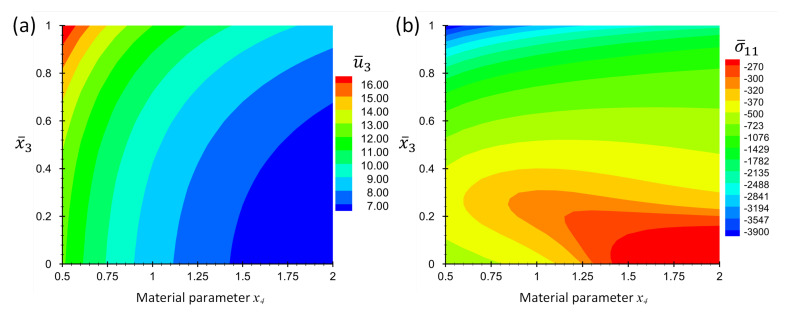
Contour plots of (**a**) non-dimensional displacement, ${\bar{u}}_{3}$, and (**b**) non-dimensional stress, ${\bar{\sigma }}_{11}$, at location, ${\bar{x}}_{1}={\bar{x}}_{2}=0.5$, in terms of ${\bar{x}}_{3}$ and $x_{4}$ for Example 2 for thickness ratio $L_{x}/L_{z}=4$.

**Figure 7:**
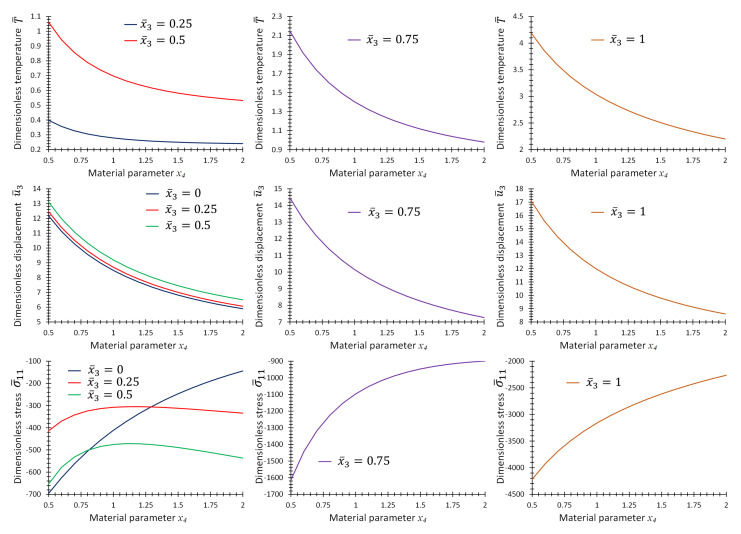
Graphs of non-dimensional quantities $\bar{T}$, ${\bar{u}}_{3}$ and ${\bar{\sigma }}_{11}$, at location, ${\bar{x}}_{1}={\bar{x}}_{2}=0.5$, for different ${\bar{x}}_{3}$ in terms of material parameter $x_{4}$ for Example 2 for thickness ratio $L_{x}/L_{z}=4$.

Figure 6 shows the contour plots of non-dimensional displacement, ${\bar{u}}_{3}$, and stress ${\bar{\sigma }}_{11}$ for the points ${\bar{x}}_{1}={\bar{x}}_{2}=0.5$ and ${\bar{x}}_{3}\in \left[0,1\right]$ in terms of material parameter, *p*. The color legend of this figure shows the numeric values, but to make it easier for readers to extract accurate numerical values, some graphs are also added in Figure 7. The graphs of $\bar{T}$, ${\bar{u}}_{3}$ and ${\bar{\sigma }}_{11}$ in terms of *p* are given in Figure 7 at some specific ${\bar{x}}_{3}$ values while ${\bar{x}}_{1}={\bar{x}}_{2}=0.5$.

### 5.3. Example 3

In the present example, more complex boundary conditions and also material distributions are considered. The aim of this example is to show the potential of the PGD technique when dealing with such complexities. The boundary conditions regarding the elasticity problem on the four peripheral surfaces of the domain are clamp-free-symmetric-free, which are defined in detail as follows:

(38) $$\begin{array}{cc} u_{1}=u_{2}=u_{3}=0,\;\; & \mathrm{on}\;\mathrm{face}\;\;{\bar{x}}_{1}=0\;,\\ u_{1}=0,\;\; & \mathrm{on}\;\mathrm{face}\;\;{\bar{x}}_{1}=1\;. \end{array}$$

Note that Equation (38) shows the essential (or Dirichlet) boundary conditions related to the elasticity equations. Zero traction is considered on the natural (or traction) boundaries.

Regarding the thermal boundary conditions, zero temperature is enforced on the two faces ${\bar{x}}_{1}=0$ and ${\bar{x}}_{1}=1$ as Dirichlet boundaries. A constant heat flux of q=1000 is applied on upper face of the domain where ${\bar{x}}_{3}=0$. All the other faces are considered insulated.

**Figure 8:**
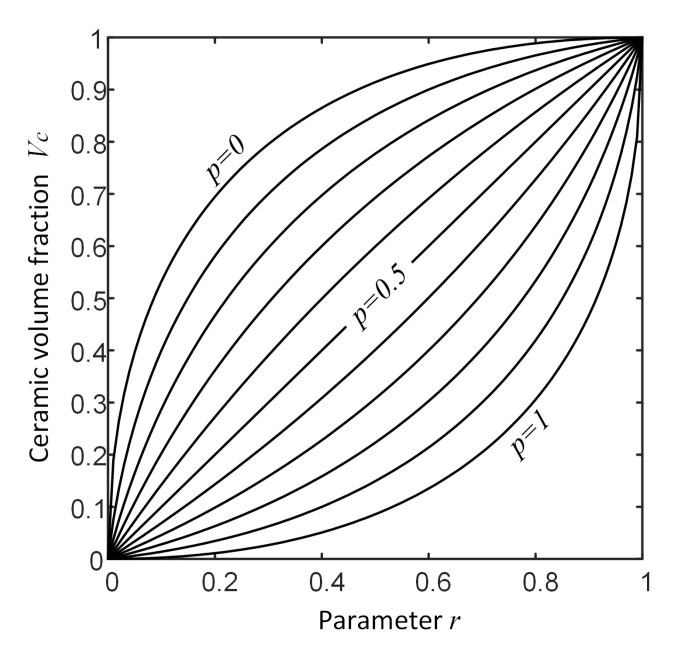
Ceramic volume fraction as function of controlling parameters *p* and *r* related to Equation (39).

**Figure 9:**
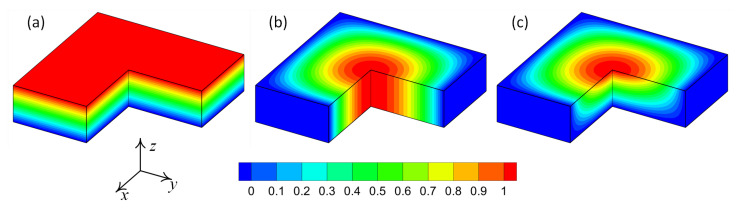
Schematic representation of ceramic volume fraction distribution: (**a**) one-directional variation; (**b**) two-directional variation; (**c**) three-directional variation.

Three different material distribution models are considered in the present example. In these models, the material gradation is considered in one, two and three directions. In each distribution model, a material parameter, *p*, controls the materials distribution. To do this, the following common formula is used to define the ceramic volume fraction, $V_{c}$:

(39) $$V_{c}\left(x\right)=\frac{-2p+2\sqrt{p^{2}+r\left(1-2p\right)}}{1-2p}+r,$$

where parameters *p* and *r* are two controlling parameters that manage the spatial distribution of the ceramic volume fraction, $V_{c}$, over the physical space. Figure 8 shows the volume fraction, $V_{c}$, as a function of two parameters *p* and *r*. In the present example, the parameter $p=x_{4}\in \left[0,1\right]$ is considered as the material distribution parameter that takes part in the parametric study. Meanwhile, the parameter *r* is considered as a known function of the physical space. In other words, the parameter *r* controls the trend of gradation of material in the physical space. In the present example, three different trends for material distribution are considered. For the first case, the material gradation is considered only in the thickness direction (one-directional variation). In the second case, the material gradation is considered plane wise (two-directional variation). Finally, the third case consists of material gradation in all directions (three-directional variation). A schematic presentation of the material distribution for one, two and three-directional material variations are shown in Figure 9.

In the one-directional material variation, the ceramic volume fraction evolves just in the thickness direction. Therefore, the parameter *r* is a function of ${\bar{x}}_{3}$. Consider the pure metallic phase at the lower face ($V_{c}=0$ at ${\bar{x}}_{3}=0$) and the pure ceramic phase at the top face ($V_{c}=1$ at ${\bar{x}}_{3}=1$); then, the parameter r(x) is defined as follows:

(40) $$r\left(x\right)={\bar{x}}_{3}.$$

For the case of two-directional material variation, assume that the material distribution is completed in two directions $x_{1}$ and $x_{2}$. In this case, assume four lateral faces are made of pure metal, and the center line of the plate is pure ceramic. In such a case, the parameter r(x) is as follows:

(41) $$r\left(x\right)=16{\bar{x}}_{1}{\bar{x}}_{2}\left(1-{\bar{x}}_{1}\right)\left(1-{\bar{x}}_{2}\right).$$

In the third case, assume the material evolves in all three directions $x_{1}$, $x_{2}$ and $x_{3}$. Consider all four lateral sides of the plate and also the bottom face consist of pure metal. However, the center point at the upper face consists of pure ceramic. The parameter r(x) is as follows:

(42) $$r\left(x\right)=16{\bar{x}}_{1}{\bar{x}}_{2}{\bar{x}}_{3}\left(1-{\bar{x}}_{1}\right)\left(1-{\bar{x}}_{2}\right).$$

All three cases are solved using the proposed PGD technique in 4D computational space $\left(x_{1},x_{2},x_{3},x_{4}\right)$, where $x_{4}=p$. The number nodes in each direction is selected here as 61, 61, 41 and 21, respectively, in directions $x_{1}$ to $x_{4}$. Both thermal and elasticity problems are solved considering 30 enrichment steps.

**Figure 10:**
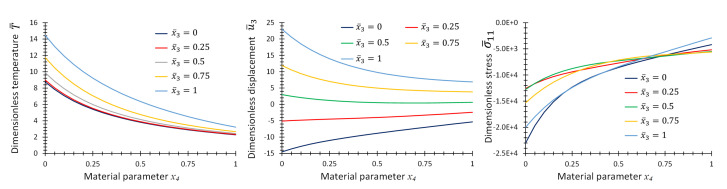
Graphs of $\bar{T}$, ${\bar{u}}_{3}$ and ${\bar{\sigma }}_{11}$ as functions of material parameter $x_{4}$ at location, ${\bar{x}}_{1}={\bar{x}}_{2}=0.5$, and different ${\bar{x}}_{3}$ for Example 3 for one-directional material variation.

**Figure 11:**
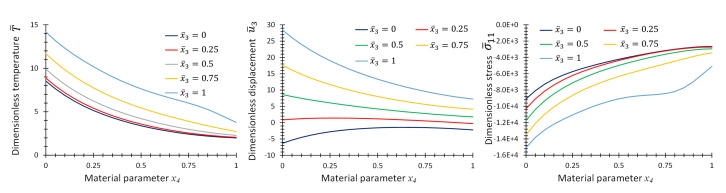
Graphs of $\bar{T}$, ${\bar{u}}_{3}$ and ${\bar{\sigma }}_{11}$ as functions of material parameter $x_{4}$ at location, ${\bar{x}}_{1}={\bar{x}}_{2}=0.5$, and different ${\bar{x}}_{3}$ for Example 3 for two-directional material variation.

**Figure 12:**
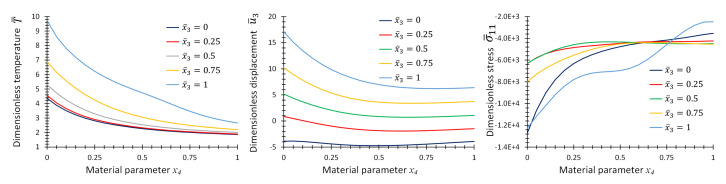
Graphs of $\bar{T}$, ${\bar{u}}_{3}$ and ${\bar{\sigma }}_{11}$ as functions of material parameter $x_{4}$ at location ${\bar{x}}_{1}={\bar{x}}_{2}=0.5$, and different ${\bar{x}}_{3}$ for Example 3 for three-directional material variation.

Figure 10 provides the graphs of non-dimensional temperature, $\bar{T}$, displacement, ${\bar{u}}_{3}$, and stress, ${\bar{\sigma }}_{11}$, as functions of material parameter, *p*, at some specific points on the center line of the plate for the first case of material variation. Figure 11 and Figure 12 provide the same graphs considering the second and third case of material variation, respectively.

### 5.4. Example 4

**Figure 13:**
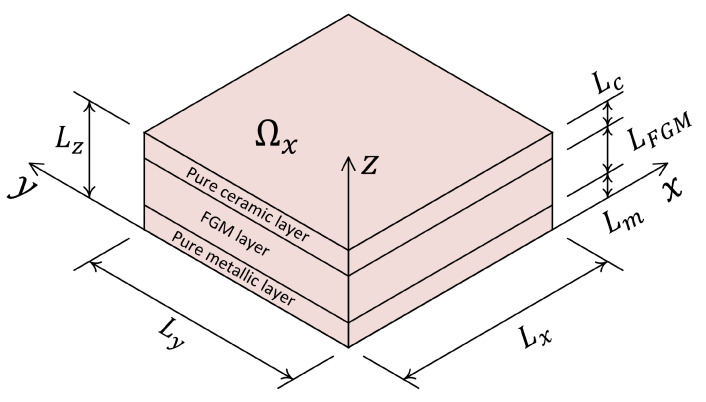
Schematic representation of a layered composite FGM plate considered for Example 4.

In the last example, a layered FGM composite plate with three layers is considered. A schematic of the composite layup is shown in Figure 13. As shown in this figure, the lower layer is pure metallic, the top layer is pure ceramic and the middle layer is the transition zone or FGM layer. The thicknesses of the metallic, ceramic and FGM layers are $L_{m}$, $L_{c}$ and $L_{FGM}$, respectively.

A parametric analysis is conducted here consisting two material parameters. The first material parameter, $p_{1}=x_{4}$, describes the thickness of the bottom and top layers. The thicknesses of these two layers are considered the same and are given as $L_{m}=L_{c}=p_{1}L_{z}$. While the total plate thickness, $L_{z}$, does not change, the thickness of the FGM layer is $L_{FGM}=\left(1-2p_{1}\right)L_{z}$.

The second material parameter, $p_{2}=x_{5}$, represents the distribution of constituents in the FGM layer. Here, the distribution of ceramic volume fraction, $V_{c}$, through the middle layer is considered as given in Equation (39). The controlling parameter, *p*, in Equation (39) is selected as $p=p_{2}$ and the function *r* is defined as follows:

(43) $$r\left(x\right)=\frac{{\bar{x}}_{3}-p_{1}}{1-2p_{1}}\;,\;\;\;\;p_{1}\leq {\bar{x}}_{3}\leq \left(1-p_{1}\right)\;.$$

**Figure 14:**
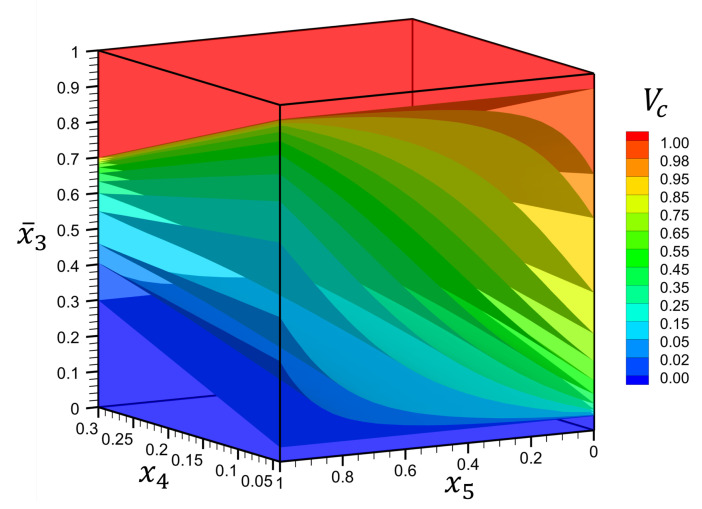
Isovalue surfaces of ceramic volume fraction, $V_{c}$, through the thickness, ${\bar{x}}_{3}$, as function of material parameters $x_{4}$ and $x_{5}$ for Example 4.

The physical domain is selected as a square plate, $L_{x}=L_{y}$, with a thickness ratio of $L_{x}/L_{z}=4$. The range of material parameters is selected as $0.04\leq p_{1}\leq 0.3$ and $0\leq p_{2}\leq 1$. To have a better understanding of the above-mentioned material distribution, see Figure 14. In this figure, the isovalue surfaces of ceramic volume fraction, $V_{c}$, are shown in terms of $p_{1}$, $p_{2}$ and ${\bar{x}}_{3}$.

In the present example, the parametric space, $\Omega_{p}\in R^{2}$, is a two-dimensional space. Therefore, the computational space, $\Omega \in R^{5}$, is a five-dimensional space. Solving such a problem using classical mesh based methods is computationally intensive because a full 3D thermo-elastic analysis must be repeated for any combinations of material parameters $p_{1}$ and $p_{2}$. Whereas, using the PGD technique, the original 5D problem reduces to a set of 1D problems. This reduction of the problem complexity implies significant computational cost reduction.

All boundary conditions are the same as Example 2 in Section 5.2. Four peripheral faces are simply supported as described in Equation (38). The top face is under a uniform heat flux as given in Equation (37), while all other faces are maintained at zero temperature.

The number of nodes in each direction is selected here as 61, 61, 51, 14 and 16, respectively, in the directions $x_{1}$, $x_{2}$, $x_{3}$, $p_{1}$ and $p_{2}$. Both thermal and elasticity problems are solved considering 60 enrichment steps.

**Figure 15:**
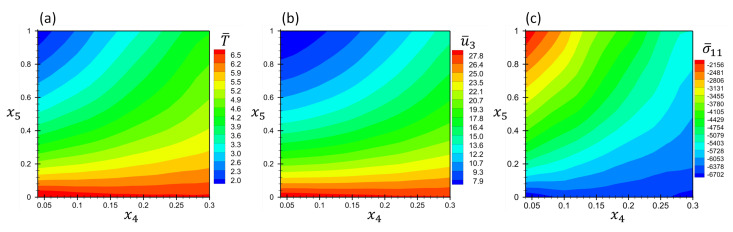
Contours of non-dimensional quantities (**a**) $\bar{T}$, (**b**) ${\bar{u}}_{3}$ and (**c**) ${\bar{\sigma }}_{11}$ as function of material parameters $x_{4}$ and $x_{5}$ at the location $\left({\bar{x}}_{1},{\bar{x}}_{2},{\bar{x}}_{3}\right)=\left(0.5,0.5,1\right)$ for Example 4.

To represent the results, the contours of non-dimensional field variables at location $\left({\bar{x}}_{1},{\bar{x}}_{2},{\bar{x}}_{3}\right)=\left(0.5,0.5,1\right)$ are plotted in Figure 15 in terms of material parameters $p_{1}$ and $p_{2}$. The temperature, displacement and stress fields are normalized using Equation (36).

## 6. Conclusions

In the present work, the PGD was used as an a priori model order reduction technique to solve a high-dimensional parametric problem by reducing it to a set of one-dimensional problems. This approach avoids repeated simulations to perform parametric analysis. Therefore, this technique overcomes the curse of dimensionality and makes it possible to deal with problems consisting of many parameters, whereas traditional grid-based techniques fail due to computational costs. The thermo-elastic analysis of FGM thick plates was considered here because of the high importance of such smart materials in industrial applications and also the high importance of parametric analysis in the accurate (or optimum) design of such materials. In addition, multi-directional material properties variation was considered here to address very recent technologies regarding the additive manufacturing of FGM materials. Accuracy and potential applications of the PGD technique were shown via solving some sample examples and comparing with analytical solutions when available. One proposal for extending the present work is to consider the curved FGM panels and to use the geometry mapping technique to transfer the governing equations into a regular computational domain and try to solve the problem in the computational domain.

## Figures and Tables

**Table 1:** The indices to be used in the generic form of elasticity equations given in Equation (11).

Equation	Term	a	b	c	d	e	f
Equation (4)	1	1	1	1	2	1	1
	2	1	1	2	2	1	2
	3	1	1	3	3	1	3
	4	1	2	2	1	4	4
	5	1	2	1	2	4	4
	6	1	3	1	3	6	6
	7	1	3	3	1	6	6
Equation (5)	1	2	1	2	1	4	4
	2	2	1	1	2	4	4
	3	2	2	1	1	1	2
	4	2	2	2	2	2	2
	5	2	2	3	3	2	3
	6	2	3	3	2	5	5
	7	2	3	2	3	5	5
Equation (6)	1	3	1	1	3	6	6
	2	3	1	3	1	6	6
	3	3	2	3	2	5	5
	4	3	2	2	3	5	5
	5	3	3	1	1	1	3
	6	3	3	2	2	2	3
	7	3	3	3	3	3	3

**Table 2:** Material properties of metallic (Monel) and ceramic (Zirconia) phases [29].

	Monel	Zirconia	
Bulk modulus (κ)	227.24	125.83	GPa
Shear modulus (μ)	65.55	58.077	GPa
Thermal expansion (α)	$15\times 10^{-6}$	$10\times 10^{-6}$	1/K
Thermal conductivity (*k*)	25.0	2.09	W/mK

**Table 3:** Non-dimensional quantities $\bar{T}$, ${\bar{u}}_{3}$ and ${\bar{\sigma }}_{11}$ for Example 1 at location ${\bar{x}}_{1}={\bar{x}}_{2}=0.5$ and different ${\bar{x}}_{3}$ for present and reference solutions; the values in parentheses show relative percentage error.

	${\bar{x}}_{3}$	$L_{x}/L_{z}=4$		$L_{x}/L_{z}=10$	
		**Reference [29]**	Present *	**Reference [29]**	Present *
$\bar{T}$	0.5	0.2101	0.2105 (0.2)	0.2432	0.2436 (0.2)
	1	3.043	3.049 (0.2)	6.021	6.010 (0.2)
${\bar{u}}_{3}$	0.5	2.143	2.147 (0.2)	5.635	5.623 (0.2)
	0	1.901	1.904 (0.2)	5.522	5.510 (0.2)
	1	−1018	−1024 (0.6)	−1006	−1014 (0.8)
${\bar{\sigma }}_{11}$	0.5	−204.8	−205.0 (0.1)	243.0	−243.6 (0.2)
	0	−73.53	−73.76 (0.3)	−75.78	−75.09 (0.9)

## Data Availability

No new data were created.

## Appendix A. Separated Representation of a Known Function

Assume g(x) is a known multivariate function on the space $\Omega \in R^{N_{D}}$. The objective is to decompose (or to separate) the function g(x) to a set of univariate functions as follows:

(A1) $$g\left(x\right)=\sum_{i=1}^{N_{g}}\prod_{j=1}^{N_{D}}g_{ji}\left(x_{j}\right)$$

Each function $g_{ji}\left(x_{j}\right)$ can be approximated using a set of approximation functions, $M_{j}\left(x_{j}\right)$, and corresponding coefficients, $g_{ji}$, as follows:

(A2) $$g\left(x\right)=\sum_{i=1}^{N_{g}}\prod_{j=1}^{N_{D}}M_{j}^{T}\left(x_{j}\right)g_{ji}$$

Without loss of generality, consider that the terms i=1 to i=(n−1) were calculated before and we are searching for the next term i=n. In this case, the residual (or error) of the terms i=1,2,⋯,(n−1) is:

(A3) $$r^{n-1}\left(x\right)=g\left(x\right)-\sum_{i=1}^{n-1}\prod_{j=1}^{N_{D}}M_{j}^{T}\left(x_{j}\right)g_{ji}$$

Now, the next term, *n*, should to be added to the existing n−1 terms to eliminate (or reduce) this error. In other words, the term i=n must be found in such a way that we approximate the error function $r^{n-1}\left(x\right)$. Therefore, we have:

(A4) $$\prod_{j=1}^{N_{D}}M_{j}^{T}\left(x_{j}\right)g_{jn}\approx r^{n-1}\left(x\right)$$

A least square sense can be followed to calculate the coefficients vector $g_{jn}$ by minimizing the following error norm:

(A5) $$∥e∥=\int_{\Omega }{\left(\prod_{j=1}^{N_{D}}M_{j}^{T}\left(x_{j}\right)g_{jn}-r^{n-1}\left(x\right)\right)}^{2}d\Omega$$

The first derivatives of the error norm ∥e∥ with respect to $g_{jn}$ must be vanished to minimize ∥e∥. In other words:

(A6) $$\frac{\partial ∥e∥}{\partial g_{dn}^{\alpha }}=0,\;\;\;d=1,2,...,N_{D},\;\;\;\alpha =1,2,...,m$$

where $g_{dn}^{\alpha }$ is the α-th element of the unknown vector $g_{dn}$. *m* is the number of approximation functions in $M_{d}\left(x_{d}\right)$. Expand Equation (A6) as follows:

(A7) $$\begin{array}{c} \frac{\partial ∥e∥}{\partial g_{jn}^{\alpha }}=0\Rightarrow \int_{\Omega }M_{d}^{\alpha }\left(M_{d}^{T}g_{dn}\right)\prod_{\begin{array}{c} j=1\\ j\neq d \end{array}}^{N_{D}}{\left(M_{j}^{T}g_{jn}\right)}^{2}d\Omega =\\ \int_{\Omega }M_{d}^{\alpha }\prod_{\begin{array}{c} j=1\\ j\neq d \end{array}}^{N_{D}}\left(M_{j}^{T}g_{jn}\right)r^{n-1}\left(x\right)d\Omega \end{array}$$

where $M_{d}^{\alpha }$ is the α-th member of the vector $M_{d}$ or α-th approximation function in terms of $x_{d}$. Separate the integrals in Equation (A7) and rearrange it as follows:

(A8) $${\left(\int M_{d}^{\alpha }M_{d}dx_{d}\right)}^{T}g_{dn}=\frac{\int M_{d}^{\alpha }dx_{d}\prod_{\begin{array}{c} j=1\\ j\neq d \end{array}}^{N_{D}}\int M_{j}^{T}g_{jn}dx_{j}}{\prod_{\begin{array}{c} j=1\\ j\neq d \end{array}}^{N_{D}}\int {\left(M_{j}^{T}g_{jn}\right)}^{2}dx_{j}}$$

Remember that $d=1,2,...,N_{D}$ and α=1,2,...,m. Therefore, Equation (A8) consists of a set of nonlinear algebraic equations for the unknown coefficients $g_{dn}^{\alpha }$. This system of equations can be solved using the fixed-point iterative approach. To do this, assume that the coefficients are unknown in one direction, while they are known in other directions. This linearizes the system of equations and makes it possible to use a direct solver to find the unknown coefficients. Next, assume another direction as the unknown and repeat the process until satisfying a convergence criterion.

The above algorithm gives the *n*-th term of the separated representation in Equation (A2). The algorithm can be repeated, obtaining subsequent terms one by one. This process is known as the enrichment process. A stopping criterion is necessary to stop the enrichment process and limit the number of terms.

Detailed explanations and other algorithms for obtaining the separated representation of a known multivariate function are proposed in the literature, e.g., [23,26].
